# Domestication of the neotropical tree *Chrysophyllum cainito* from a geographically limited yet genetically diverse gene pool in Panama

**DOI:** 10.1002/ece3.948

**Published:** 2014-01-28

**Authors:** Jennifer J Petersen, Ingrid M Parker, Daniel Potter

**Affiliations:** 1 Department of Plant Sciences, University of CaliforniaOne Shields Avenue, Davis, California, 95616; 2 Department of Ecology and Evolutionary Biology, University of CaliforniaSanta Cruz, California, 95064; 3 Smithsonian Tropical Research InstituteApartado, 0843-03092, Balboa, Republic of Panama

**Keywords:** caimito, fruit trees, genetic diversity, Mesoamerica, Sapotaceae, semidomesticates

## Abstract

Species in the early stages of domestication, in which wild and cultivated forms co-occur, provide important opportunities to develop and test hypotheses about the origins of crop species. *Chrysophyllum cainito* (Sapotaceae), the star apple or caimito, is a semidomesticated tree widely cultivated for its edible fruits; it is known to be native to the neotropics, but its precise geographic origins have not been firmly established. Here, we report results of microsatellite marker analyses supporting the hypothesis that the center of domestication for caimito was the Isthmus of Panama, a region in which few crop species are believed to have originated, despite its importance as a crossroads for the dispersal of domesticated plants between North and South America. Our data suggest that caimito was domesticated in a geographically restricted area while incorporating a diverse gene pool. These results refute the generally accepted Antillean origin of caimito, as well as alternative hypotheses that the species was domesticated independently in the two areas or over a broad geographic range including both. Human-mediated dispersal from Panama to the north and east was accompanied by strong reductions in both genotypic and phenotypic diversity. Within Panama, cultivated and wild trees show little neutral genetic divergence, in contrast to striking phenotypic differentiation in fruit and seed traits. In addition to providing a rare example of data that support the hypothesis of a narrow geographic origin on the Isthmus of Panama for a now widespread cultivated plant species, this study is one of the first investigations of the origins of an edible species of the large pantropical family Sapotaceae.

## Introduction

Crop domestication is a long-term evolutionary process whereby wild plant populations become exploited via human management practices, leading to phenotypic and/or genetic differences between domesticated forms and their wild progenitors. Differentiation between domesticated and undomesticated races occurs along a continuum from minor, as in the case of incipient domesticates, to extreme, as in the case of full domesticates (see definitions in Clement 1999). The trajectory of domestication may occur at the intra-or interspecific level, and may result in changes in morphological traits and the genetic loci that control them (reviewed in Pickersgill 2007) and/or allele frequencies at neutral loci (see Casas et al. 2007). The process by which human selection of natural variation over many generations may eventually result in the fixation of desired phenotypes has provided a cornerstone for expanding our understanding of evolution since Darwin's *On the Origin of Species* (Darwin 1859). In recent years, the use of molecular markers to study cultivated species and their wild relatives has revolutionized our ability to address questions about the process of domestication, including the number and locations of geographic areas that gave rise to domesticates, the effects of human selection on genetic diversity, and the relationship between genotypic and phenotypic variation. Such studies shed light on the spatial dynamics of human influences on gene pools through selection and dispersal, and they can inform policies aimed at conserving wild and crop diversity and promoting regional food security.

Tropical America has long been recognized as the region of origin for many domesticated plant species, ranging from minor cultigens with local significance only to some of the world's most important crops. Current evidence indicates that neotropical domesticates come from a broad range of ecological habitats, but seasonal lowland neotropical forests are exceptional in terms of the number of species that have originated there (Ranere et al. 2009; Piperno 2011). Mesoamerica has been identified as one of three specific areas of independent plant domestication in the neotropics (Pickersgill 2007). The region is an important center of cultural and crop diversity, with over 5000 species of plants utilized by local cultural groups (Casas et al. 1994). The area that is now Mexico is considered to be the source of the majority of crop plants of Mesoamerican origin (Ranere et al. 2009). Farther to the south, the Isthmus of Panama was for centuries an important crossroad for exchange of cultivated plants between the Mexican center of crop origin and the Andean and Amazonian regions in South America (Dickau et al. 2007), but to date, only one herbaceous domesticate, *Cucurbita moschata* Duchesne (Merrick 1995; Piperno and Pearsall 1998), and one cultivated tree, *Chrysophyllum cainito* L. (Petersen et al. 2012), have been suggested to have Panamanian origins. Here, we use microsatellite marker data to further investigate the origins and spread of the second of these species, commonly known as “star apple” or “caimito,” a tropical fruit tree in the early stages of domestication (Parker et al. 2010).

*Chrysophyllum cainito* (Sapotaceae) is a tree that grows to 25 m tall and is esteemed for its edible fruit and ornamental value. The fruits are high in polyphenolic antioxidants (Luo et al. 2002; Parker et al. 2010) and when ripe have a unique flavor/fragrance profile that may be due to terpenoids (Pino et al. 2002). The ornamental value of the species is enhanced by the presence of a dense indumentum of gold-colored hairs on the undersides of the leaves, as described by the generic name. Chromosome counts summarized by Johnson (1991) suggest that the species is a diploid with 2*n* = 24 (Krishnaswamy & Rahman 1949) or 26 chromosomes (Tjio 1948; Miège 1954). Small bees such as *Tetragonisca spp*. pollinate the flowers (Parker et al. 2010). Caimito is reported to be self-fertile (Crane 2008), although natural populations would be expected to show high levels of outcrossing if they behave like most tropical tree species (Ward et al. 2005).

Historical records from European plant explorers dating from the 16th and 17th centuries indicate that *Chrysophyllum cainito* has long been cultivated in the neotropics, from the Greater and Lesser Antilles to Panama (Seeman 1852; see references cited in Petersen et al. 2012). The species is presently cultivated in the Antilles, Mesoamerica, and South America, as well as in parts of Florida, Hawaii, and South-East Asia (Morton 1987; Crane 2008). In many areas in Mesoamerica and the Caribbean, caimito is a common backyard tree; the fruits are often sold in markets, but large-scale production does not occur (Chízmar-Fernández et al. 2009). The trees are propagated by seed. *Chrysophyllum cainito* can be found growing in both wild and cultivated settings in Panama, Jamaica, and the Dominican Republic (I. M. Parker, J. J. Petersen, & D. Potter, pers. obs.). Based on our observations of significant differences in fruit phenotypic traits between wild and cultivated trees in Panama (Parker et al. 2010) and the fact that wild and cultivated forms of the species coexist, we concluded that caimito fits Clement's (1999) definition of a semidomesticated plant, that is, one exhibiting significant modification due to human selection but not dependent on human intervention for survival.

Although *Chrysophyllum cainito* is known to be native to the neotropics, its precise native range and area(s) of domestication have not been established with certainty. The prevailing hypothesis for many years was that the species is native to the Greater Antilles and has naturalized into Central and South America, based in part on the widespread use of the Taino name “caimito” (Standley and Williams 1967; Pennington 1990). An alternative hypothesis is that caimito is native to the Isthmus of Panama and was domesticated there. Sloane (1725; citation in De Candolle 1884) considered that putative wild trees on Jamaica were escapes from cultivation, and there are historical observations that both wild and cultivated caimito were present on the Isthmus of Panama and the surrounding islands (i.e., Seeman 1852). More recent support for a Panamanian origin came from our recent phylogeographic and phylogenetic analyses of DNA sequences, which showed greater haplotype diversity within *C. cainito* in the southern part of its range and indicated that its closest relative is a clade containing *C. argenteum* subsp. *panamense*, a taxon from southern Central and South America (Petersen et al. 2012). Finally, comparisons of wild and cultivated trees in Panama revealed a clear signature of domestication in seed and fruit traits; wild trees had significantly smaller, more acidic fruits with lower concentrations of sugars, and higher concentrations of phenolics than those of cultivated trees (Parker et al. 2010). In contrast, the fruits of all of the trees we encountered in the Greater Antilles, whether growing wild or under cultivation, had larger fruits like those with the putative domesticated phenotype in Panama (Petersen et al. 2012).

Aside from the Antillean hypothesis and the Panama Isthmus hypothesis, additional possibilities include independent domestications in these two areas or multiple domestications over a broad geographic range. The latter result might be expected, because hypotheses of multiple domestications have been supported for many other cultivated species, and a pattern of diffuse origins is especially common among perennial fruit crops (Miller and Gross 2011).

The objectives of the current study were to test competing hypotheses for the origins of wild and domesticated forms of caimito and to explore patterns of genetic diversity and structure of cultivated and wild trees in southern Mesoamerica (Panama, Costa Rica), northern Mesoamerica (Guatemala, Mexico), and the Antilles (Jamaica and the Dominican Republic). We chose to study the origins and domestication of *C. cainito* for three primary reasons. First, working with a semidomesticate allows us to explore the relationships among phenotypic variation, genetic diversity, and cultivation status within a single species. Second, caimito is a member of the chicle family, Sapotaceae, a large, pantropical group with many ecologically and economically important species (e.g., sapodilla, mamey, shea, and gutta-percha) that are cultivated for their edible fruits and/or their latex, but despite the importance of domestication in the family, very little is known about patterns of anthropogenic evolution in this group. Third, in spite of uncertainty about its origins, caimito has been used as a native species in reforestation projects in Panama; thus, clarifying the native range and domestication status of the species has important conservation implications.

In this study, we addressed the following specific questions using highly variable microsatellite marker data:

1. What are the origins of extant wild and cultivated populations of *C. cainito*? Is genetic diversity greater in southern Mesoamerica than in northern Mesoamerica and the Antilles, indicating that the species is native to Panama and was domesticated there, while wild populations in the Antilles represent escapes from cultivation?
2. How has human selection affected levels of genetic diversity in cultivated caimito? Do cultivated trees in the area(s) of origin demonstrate a strong genetic bottleneck driven by anthropogenic selection of a limited number of genotypes, or, alternatively, did the initial stages of the domestication process incorporate high levels of genetic diversity? Is there marked differentiation between wild and cultivated gene pools within Panama?

## Materials and Methods

### Field sampling

We sampled 206 individuals of *Chrysophyllum cainito* collected from localities in the Greater Antilles (Dominican Republic and Jamaica) and both northern (Mexico and Guatemala) and southern (Costa Rica and Panama) Mesoamerica, as well as a total of 26 individuals of the congeneric species *C*. *argenteum*,*C. mexicanum,* and *C. oliviforme* from areas where they were growing in proximity to *C. cainito* (Table 1).

**Table 1:** Locality information and sample size of cultivated and wild *Chrysophyllum cainito* and close relatives.

Collection number	Collection locality	Code	*N*	Latitude	Longitude
*C. cainito*, NORTH, CULTIVATED					
Dominican Republic					
JP760	Cambita Garabitos, San Cristóbal	GAR	1	18°27.262 N	70°11.866 W
JP821	Camu, Puerto Plata	CAM	2	19°41.643 N	70°37.500 W
JP813	Cotui-Maimon, Sanchez Ramirez	COT	4	19°01.567 N	70°09.023 W
JP797	Cruce de Cenovi, La Vega	CEN	1	19°12.478 N	70°20.976 W
JP802	El Caimito, Duarte	CAI	5	19°10.141 N	70°17.474 W
JP827	Gaspar Hernandez, Espaillat Salcedo	GAS	1	19°37.774 N	70°16.389 W
JP800	La Bandera, Duarte	BAN	1	19°11.505 N	70°19.312 W
JP829	La Vega, La Vega	VEG	1	19°14.729 N	70°32.287 W
JP828	Moca, Espaillat Salcedo	MOC	1	19°25.122 N	70°30.218 W
JP811	Pimentel, Duarte	PIM	3	19°12.997 N	70°07.394 W
JP796	Puente de Blanco, Monsenor Nouel	BLA	1	19°01.183 N	70°27.062 W
JP768	Villa Mella-Yamasa, Santo Domingo	MEL	3	18°37.069 N	69°56.186 W
JP773	Yamasá, Monte Plata	YAM	6	19°10.531 N	70°17.265 W
JP774	Yamasá-cacao, Monte Plata	YAC	4	18°47.237 N	70°00.981 W
JP823	Yásica Abajo, Puerto Plata	YAS	3	19°38.137 N	70°35.788 W
Guatemala					
GUA08-7	Benque, El Amatillo, Izabal	BEN	1	15°32.577 N	88°54.555 W
GUA08-1	La Ribosa, Izabal	RIB	1	15°26.370 N	88°57.214 W
GUA08-8	Puerto Barrios, Izabal	BAR	2	15°32.324 N	88°44.372 W
GUA08-2	Rio Dulce, Izabal	DUL	1	15°39.253 N	89°00.500 W
GUA07-1	Salamá, Baja Verapaz	SAL	3	15°05.590 N	90°15.570 W
GUA08-6	San Felipe, Izabal	FEL	1	15°38.215 N	89°00.030 W
GUA08-3	Santa Herminia, Izabal	HER	3	15°38.197 N	88°59.720 W
Jamaica					
JP135	Albert Town, Trelawny Parish	ALB	1	18°17.340 N	77°32.594 W
JP104	Elderski, Elderski District	ELD	3	18°13.776 N	77°48.027 W
JP101	Johnson, St. James Parish	JOH	2	18°15.708 N	77°49.755 W
JP128	Kinloss-Clark Town Road, Trelawny Parish	KIN	1	18°24.157 N	77°33.716 W
JP119	Marshal's Pen, Manchester Parish	MAR	1	18°03.608 N	77°31.822 W
JP124	Mountainside, St. Elizabeth Parish	MOU	4	17°59.415 N	77°44.760 W
JP100	Newton, St. Elizabeth Parish	NEW	1	18°07.543 N	77°44.879 W
JP103	Niagra River, St. Elizabeth Parish	NIA	1	18°14.744 N	77°48.489 W
JP98	Scott's Pass, Clarendon Parish	SCO	2	18°00.588 N	77°23.000 W
JP134	Ulster Springs, Trelawny Parish	ULS	1	18°19.174 N	77°31.180 W
Mexico					
JP253	Bacalar, Quintana Roo	BAC	3	18°41.092 N	88°23.483 W
JP90	Cooperativa Emilano Zapato, Yucatán	COO	1	20°13.876 N	88°20.324 W
JP618	Ejido 20 de Noviembre, Campeche	EJI	1	18°27.183 N	89°18.335 W
JP93	Maní, Yucatán	MAN	1	20°23.242 N	89°23.181 W
JP830	Martínez de la Torre, Veracruz	MAR	1	20°03.220 N	97°03.420 W
JP84	Muna, Yucatán	MUN	1	20°29.800 N	89°42.719 W
JP675	Narciso Mendoza, Campeche	MEN	3	18°13.878 N	89°27.330 W
JP91	Oxkutzcab, Yucatán	OXK	2	20°17.549 N	89°25.147 W
JP89	Santa Elena, Yucatán	ELE	1	20°19.438 N	89°38.643 W
JP94	Tecoh, Yucatán	TEC	1	20°44.513 N	89°28.344 W
JP85	Tikul, Yucatán	TIK	4	20°23.768 N	89°32.738 W
JP75	Valladolid, Yucatán	VAL	4	20°41.782 N	88°12.253 W
JP77	Xocen, Yucatán	XOC	1	20°35.927 N	88°09.760 W
JP81	Yaxcoba, Yucatán	YAX	3	20°36.011 N	88°48.891 W
*C. cainito,* NORTH, WILD					
Dominican Republic					
JP818	Altamira	ALT	1	19°40.553 N	70°49.779 W
JP759	La Colonia, San Cristóbal	COL	1	18°29.344 N	70°14.795 W
JP798	Cruce de Cenovi, La Vega	CRU	1	19°12.478 N	70°20.976 W
Jamaica					
JP95	Cave Valley, St. Ann Parish	CAV	2	18°12.869 N	77°22.695 W
JP107	Ipswich/Red Gate, St. Elizabeth Parish	IPS	4	18°10.588 N	77°49.963 W
JP112	Lacovia to Slipe	LAC	1	18°04.377 N	77°46.530 W
JP115	Slipe, St. Elizabeth Parish	SLI	3	18°03.533 N	77°47.133 W
JP129	Windsor Estate, Trelawny Parish	WIN	2	18°22.125 N	77°38.786 W
Mexico					
JP462	Near Valladolid, Yucatán	NVA	1	20°38.032 N	88°20.511 W
*C. cainito,* SOUTH, CULTIVATED					
Costa Rica					
JP68	Bahia Drake, Osa Peninsula	DRA	1	08°41.250 N	83°39.390 W
CR07-1	San Isidro, Perez Zeledon	PER	1	09º22.320 N	82º32.110 W
Panama					
JP228	Arraijan-Barriada 2000, Panamá	BAR	3	08°58.190 N	79°40.286 W
JP222	Arraijan-Burunga, Panamá	BUR	6	08°57.946 N	79°39.432 W
JP151	Balboa, Panamá	BAL	6	08°57.272 N	79°33.344 W
JP162	Chilibre, Panamá	CHI	14	09°11.107 N	79°36.621 W
JP177	Gamboa, Panamá	GAM	1	09°07.890 N	79°42.690 W
*C. cainito*, SOUTH, WILD					
Panama					
JP178	Camino de Cruces, Panamá	CAM	7	09°06.658 N	79°41.512 W
JP187	Clayton, Panamá	CLA	6	09°00.441 N	79°34.056 W
JP145	Ella Puru, Panamá	PUR	13	09°07.810 N	79°41.749 W
JP157	Madden, Panamá	MAD	8	09°06.906 N	79°36.945 W
JP193	Old Gamboa Rd, Panamá	OLD	7	09°06.691 N	79°41.490 W
JP207	Pipeline Road, Panamá	PIP	11	09°09.066 N	79°43.946 W
JP194	San Antonio, Panamá	ANT	5	09°07.758 N	79°41.733 W
JP197	Venta de Cruces, Panamá	VEN	8	09°07.707 N	79°41.081 W
*C. argenteum*					
Costa Rica					
JP70	Osa Peninsula	OSA	2	08°41.827 N	083°39.159 W
Dominican Republic					
JP756	La Colonia, San Cristóbal	LCO	3	18°29.344 N	70°14.795 W
JP820	Mt. Isabel de Torres, Puerto Plata	ISA	1	19°45.846 N	70°42.767 W
JP795	Mina, El Seibo	MIN	1	18°41.826 N	68°53.559 W
*C. mexicanum*					
Mexico					
JP243	Gomez Valentin Farias, Campeche	GOM	1	18°30.826 N	89°26.750 W
JP398	José María Morelos, Yucatán	MOR	1	19°44.648 N	88°42.762 W
JP73	Tres Reyes, Quintana Roo	REY	3	20.40.688 N	87°36.184 W
JP76	Xocen, Valladolid	XCE	1	20°27.878 N	88°30.339 W
*C. oliviforme*					
Dominican Republic					
JP789	El Limon, La Romana	LIM	1	18°25.732 N	68°50.589 W
JP787	Higuey, La Romana	HIG	1	18°25.732 N	68°50.589 W
JP765	Aguas Negras, Pedernales	AGU	1	18°00.439 N	71°38.799 W
JP763	Hoyo de Pelempito, Pedernales	HOY	1	18°12.000 N	71°34.000 W
JP764	Hoyo de Pelempito, Pedernales	PEL	1	18°12.000 N	71°34.000 W
JP767	Parque Nacional Sierra de Bahoruco, Pedernales	BAU	1	18°17.089 N	71°34.057 W
Jamaica					
JP120	Lincoln-Mt. Prospect, Manchester Parish	LIN	2	18°20.223 N	77°34.347 W
JP122	Shirehampton-Maidstone, Manchester Parish	SHO	2	18°50.073 N	77°35.356 W
JP130	Windsor Estate, Trelawny Parish	WES	3	18°21.159 N	77°38.714 W

Collection localities are organized by species of *Chrysophyllum*, region and cultivation status for *C. cainito* samples, and country. For each locality, a three-letter code, the number of individuals sampled (*N*), and the GPS coordinates are provided.

Our caimito collections included 125 cultivated trees from 53 localities and 81 wild trees from 17 localities (Table 1). Hereafter, we will refer collectively to the localities in the Antilles and northern Mesoamerica as the northern geographic region and to those in southern Mesoamerica as the southern geographic region (Table 1). We considered trees to be wild if they were located away from human settlements and were growing in primary or secondary forests where, to the best of our knowledge, they had not been planted. Here, “wild” refers to living in a wild habitat and does not imply ancestrally wild, or undomesticated. Trees classified as cultivated were those that occurred within areas managed by people, including backyard gardens, small-scale agricultural settings such as *ranchos*, or mixed perennial plantings such as *parcelas*. We conferred with landowners about the origins of their trees to confirm cultivation status (Table 1). Observational data on fruit phenotypes were recorded, and when trees were without fruit, we asked landowners to describe the fruit phenotype of the collected tree.

Two factors constrained our sample sizes (Table 1). In cultivated settings, trees often exist as one or two individuals in backyard gardens or other small-scale agro-ecological settings, making the circumscription of populations challenging. Wild trees in forested areas, on the other hand, are generally uncommon, widely separated, and difficult to find, rather than existing as clearly delineated stands of individuals belonging to the same population. Given these limitations, we adopted the following sampling strategy. For cultivated populations, we visited one to several households per settlement, depending on how commonly caimito trees were observed there, and we collected one to three trees per household. For wild trees, we sampled 1–13 trees at each locality, depending on the size of the population and the accessibility of the trees. Fresh leaf material was collected and dried using silica gel or used within 5 days for DNA extraction. Voucher herbarium specimens were deposited at the UC Davis Center for Biological Diversity (DAV).

### Microsatellite genotyping

Total genomic DNA was extracted from fresh or silica-dried samples using the DNeasy Plant Minikit (Qiagen, Valencia, CA). Individuals were genotyped using ten nuclear microsatellite loci developed in *C. cainito* specifically for this project (Petersen et al. 2014). Polymerase chain reactions (PCR) were carried out using forward primers fluorescently labeled with FAM, NED and HEX, and unlabeled reverse primers, as described in Petersen et al. (2014). PCR products were analyzed on an ABI prism 3100 Genetic Analyzer (Applied Biosystems, Foster City, CA) using GeneScan 400HD ROX as an internal size standard. Allele sizes were scored using GeneMapper version 3.7 (Applied Biosystems).

### Data analyses

For each geopolitical region (country), cultivation status (cultivated and wild), and geographic region (northern and southern), values were averaged over all loci to obtain the mean number of alleles (*N*_a_). Allelic richness (*A*_R_) and the number of private alleles (*P*_a_) were obtained using a rarefaction method to correct for differing population sizes in the program HP-Rare (Kalinowski 2005). We used the sign test, as suggested by Kalinowski (2004) and implemented in JMP v.9 (SAS Institute, Cary, NC), to assess the significance of differences in *A*_R_ and *P*_a_ between wild and cultivated trees in each geographic region. The population genetic structure at different geographic hierarchical levels and across management types (wild and cultivated) was investigated using analyses of molecular variance (AMOVA) (Excoffier et al. 1992; Michalakis and Excoffier 1996) in GenAlEx version 6.3 using 9999 permutations to test for significance.

We used a model-based Bayesian clustering method implemented in the program STRUCTURE, version 2.3 (Pritchard et al. 2000) to infer population structure among our samples. This method uses multilocus allele frequency data to identify *K,* the number of gene pool clusters that exist in a data set without using information about the geographic origins of the samples (using the default “no loc prior” option). We performed an initial analysis to determine the number of gene pools represented by all of our samples of *C. cainito* and related species. The entire data set, a total of 232 individuals, was analyzed by performing multiple independent runs of STRUCTURE. We used the admixture model, allowing individuals to have mixed ancestry and to fractionally assign to more than one cluster, and the correlated allele frequencies option (Falush et al. 2003). We ran 20 replicates for each value of *K* ranging from 1 to 20 with a burn-in of 100,000 generations and 200,000 Markov chain Monte Carlo (MCMC) replications. We used the program STRUCTURE HARVESTER (Earl and vonHoldt 2011) to calculate the *Ln* P(D), the estimated posterior probability of the data for a given *K* (Pritchard et al. 2000), and Δ*K*, an ad hoc value based on the second-order rate of change in the likelihood function with respect to *K* (Evanno et al. 2005). These results were used to determine the optimal *K* value and thus the highest hierarchical level of structure in the data.

We then tested for additional substructure within each gene pool cluster, including only individuals that assigned to the cluster at *q* *≥* 0.90. In order to correct for differences in individual assignments among the multiple replicate runs of each *K* value, we used the estimated cluster membership coefficient (*q*) matrices of the replicate runs of each *K* value from STRUCTURE HARVESTER (Earl and vonHoldt 2011) as input for the program CLUMPP, version 1.1.2 (Jakobsson and Rosenberg 2007), which aligns the cluster membership coefficients from multiple replicates. We used the resulting adjusted q matrices to determine which individuals were assigned at *q* *≥* 0.90 to each cluster. We then ran STRUCTURE using the same conditions as reported above to identify subclusters within each major gene pool cluster. Cluster analysis based on genetic distances among collection localities was used to examine relationships of *C. cainito* and congeners in the form of a dendrogram. We included all individuals to construct a matrix of genetic distances between all pairs of localities in the program MSAT (Minch 1997) using Cavalli-Sforza's chord distance (Cavalli-Sforza and Edwards 1967). We used this matrix to construct a neighbor-joining tree with NEIGHBOR and a majority-rule consensus tree with CONSENSE with 10,000 bootstrap replicates to indicate branch support; both programs are included in the PHYLIP software package version 3.5 (Felsenstein 1993).

## Results

### Estimates of genetic diversity

A total of 232 samples of *C. cainito* and relatives were included in this study (Table 1). Using ten microsatellite markers, we detected a total of 118 distinct alleles in wild and cultivated individuals of *C. cainito*, 41 distinct alleles in *C. argenteum* and *C. mexicanum*, and 65 distinct alleles in *C. oliviforme* (Table 2).

**Table 2:** Number of alleles observed at each of ten microsatellite loci in *Chrysophyllum cainito* and close relatives.

Locus	Repeat motif	*C. argenteum*	*C. cainito*	*C. mexicanum*	*C. oliviforme*
		Na	Na	Na	Na
G4	(AG)20	4	8	3	3
C1	(GA)12(GT)11	4	10	5	6
D9	(AG)14	6	24	4	8
G6	(AG)20	3	8	5	12
G7	(AG)18(AAG)2	2	11	4	6
E5	(AG)11	4	5	4	6
D8	(AG)14	6	11	3	6
E7	(CT)8(AC)8	3	16	6	8
F4	(AG)14	6	10	5	5
C2	(CT)15(CA)17	3	15	2	5
Total		41	118	41	65

For each locus, the microsatellite repeat motif and the number of alleles observed in each species are listed. In addition, for each species, the total number of alleles observed over all ten loci is provided.

**Figure 1:**
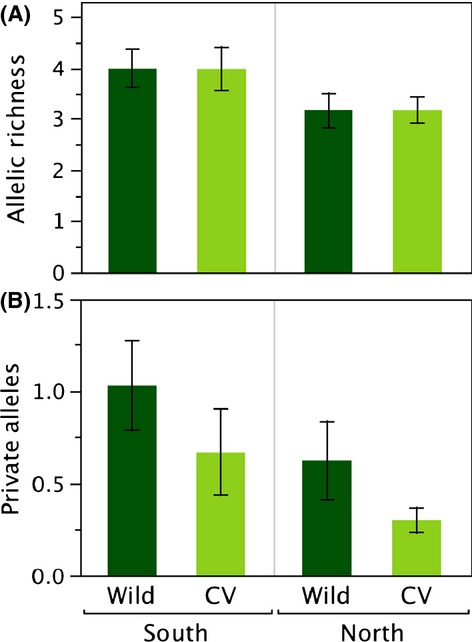
(A) Allelic richness and (B) number of private alleles for wild and cultivated (CV) trees of *Chrysophyllum cainito* in southern and northern clusters. Shown are means ± 1 standard errors across 10 microsatellite loci. Sign tests comparing north to south demonstrated significantly higher allelic richness (*P* = 0.021) and more private alleles (*P* = 0.021) in the South. Allelic richness was not significantly different between wild and cultivated trees either in the north or south (*P* > 0.3). However, private alleles were significantly more common in wild trees than cultivated trees in the south (*P* = 0.021), but not significantly different in the north (*P* > 0.7).

The mean number of alleles per locus in wild individuals of caimito collected from throughout the sample range was 10.0. Based on the STRUCTURE analysis, we divided the caimito samples into two groups; north and south (see below). Allelic richness (*A*_R_) was higher in the south than in the north (4.07 vs. 3.22, sign test *P* = 0.021; Fig. 1) as was the number of private alleles (*P*_a_), south 2.05, north 1.21, *P* = 0.021. There was no significant difference in allelic richness (*A*_R_) between wild and cultivated trees in either the south or the north (*P* > 0.3). The number of private alleles (*P*_a_) was significantly higher in wild trees in the south (*P* = 0.021) but not in the north (*P* > 0.7).

### Bayesian cluster analysis

**Figure 2:**
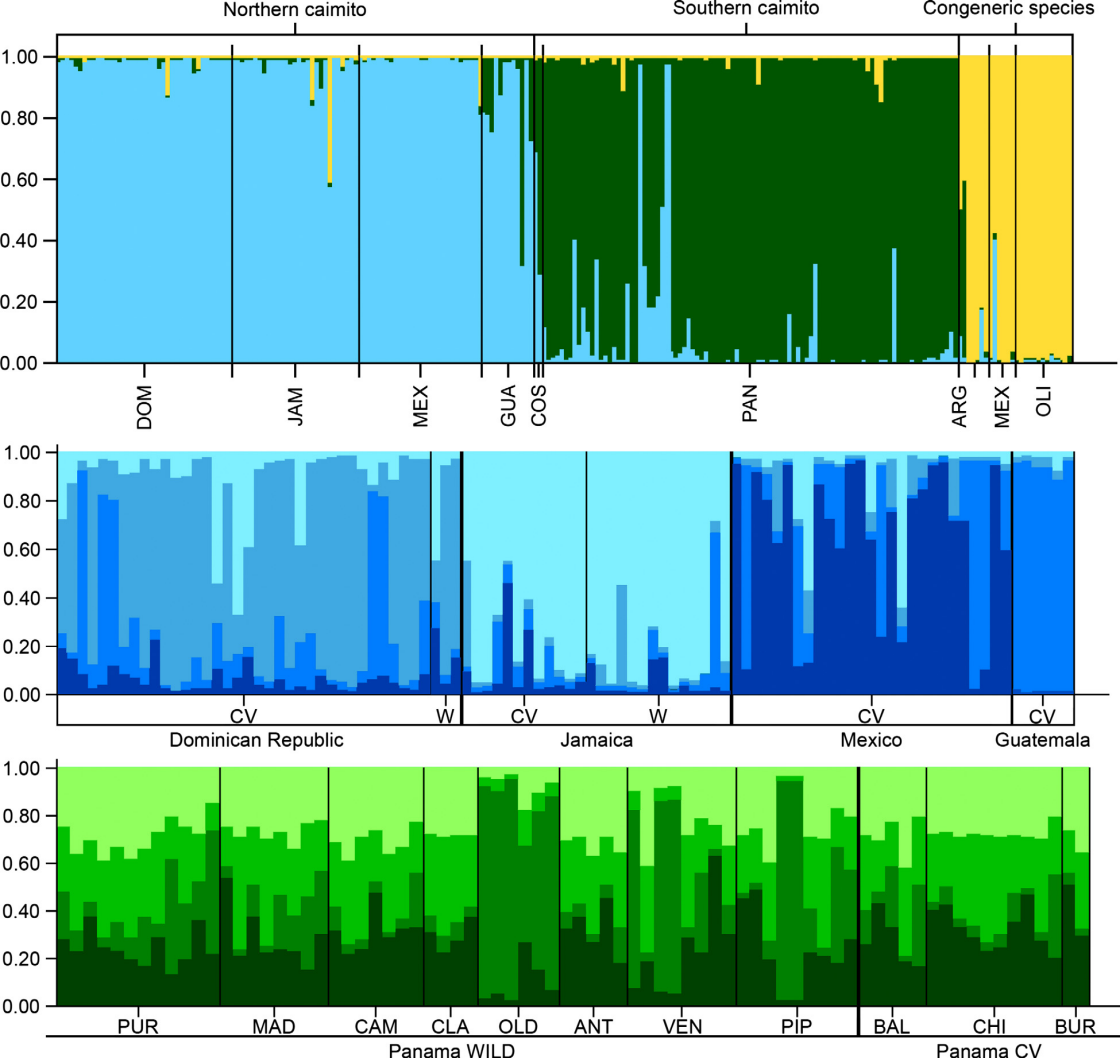
Results of Bayesian cluster analyses of genotypes at ten microsatellite loci implemented in STRUCTURE. Each bar represents an individual; each color represents a distinct gene pool cluster inferred from the analysis. Top panel: global analysis including all samples of *C. cainito* and closely related species. Middle panel: analysis including only individuals that assigned at q ≥ 0.90 to the primarily northern gene pool cluster in the global analysis. Bottom panel: analysis including only individuals that assigned at q ≥ 0.90 to the primarily southern gene pool cluster in the global analysis.

Both the Pritcenterd method (Pritcenterd et al. 2000), which examines the maximum value of ln *P*(*D*), and Evanno's Δ*K* method (2005) returned an optimal *K* value of three for our STRUCTURE analysis that included *C. cainito* individuals and three congeneric species of *Chrysophyllum*, indicating that three gene pools best explained the highest level of genetic structuring in our data set (Fig. 2). The STRUCTURE clustering algorithm assigns individuals to the inferred gene pool clusters based on an estimated membership coefficient (*q*), which measures the fraction of an individual's genome that came from each gene pool. We considered an individual to assign highly to one of the three gene pool clusters if its population membership coefficient, *q*, was ≥0.90 for that gene pool. We considered individuals whose population membership coefficients (*q*) were less than 0.90 for all gene pools to be admixed.

**Figure 3:**
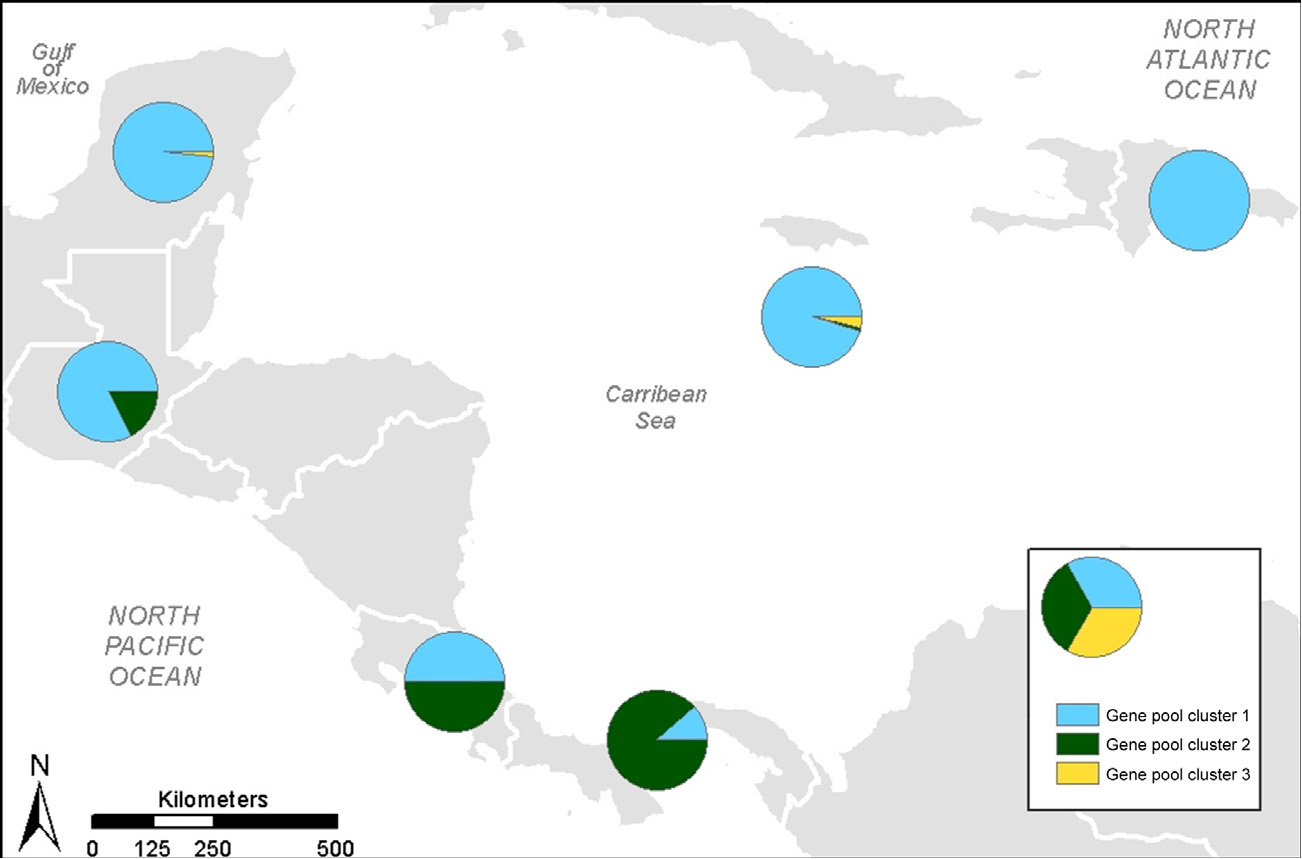
Distribution map based on STRUCTURE analysis indicating the average assignment (q) to each of the three primary gene pool clusters across all individuals sampled from each country.

The gene pool cluster to which each individual assigned was generally associated with its geographic origin (Fig. 3). The majority (92.86 and 82.50%, respectively) of wild and cultivated individuals of *C. cainito* collected from the Greater Antilles (Jamaica, the Dominican Republic) and northern Mesoamerica (Mexico and Guatemala) assigned highly to the first gene pool cluster (blue in Fig. 2), whereas the majority (77.89%) of the samples collected from Panama assigned highly to the second (green in Fig. 2). Trees sampled from Costa Rica were admixed between these two gene pools (blue and green in Fig. 2). The majority of the *C. argenteum*, (83.3%) *C. mexicanum* (57.1%), and *C. oliviforme* (100%) individuals assigned highly to the third gene pool cluster (yellow in Fig. 2). We included these other species in our analyses in order to be able to detect gene flow between them and *C. cainito* and to investigate the possibility that the cultivated and wild caimito populations with different fruit phenotypes, as observed in Panama, were as genetically distinct from one another as *C. cainito* and other taxonomically recognized species. Similar results within *C. cainito* (two gene pools consisting mostly of “northern” and “southern” individuals, respectively) were obtained when only the individuals assigned to that species were included in the analysis (data not shown).

Based on these results, we divided the caimito individuals into two major groups; northern and southern (Table 1). To test for additional levels of population structure, we conducted subsequent STRUCTURE analyses for each of the three major gene pools discussed above, in which we included only individuals that assigned highly (*q* ≥ 0.90) to the gene pool being analyzed (Fig. 2). Two individuals from Panama that assigned highly to the primarily northern gene pool cluster were excluded from this analysis. This approach revealed four subclusters within the northern gene pool cluster, each of which largely corresponded to individuals collected from a single country, with varying levels of admixture among individuals and among countries (Fig. 2). The highest levels of admixture were observed for the individuals collected in the Dominican Republic and Mexico (average highest value of *q* = 0.65 and 0.64, respectively), whereas individuals from Jamaica and Guatemala assigned more highly to individual gene pools (average highest value of *q* = 0.81 and 0.93, respectively).

All individuals that assigned highly to the southern gene pool were from Panama. As in the north, the level of substructure was best described by *K *=* *4, indicating four subclusters within the southern gene pool cluster. Most individuals exhibited genetic admixture with varying contributions from each of the four subclusters and generally low assignments (*q* < 0.50) to any one of them. The exception to this trend was observed in trees collected from three wild localities – Pipeline Road, Venta de Cruces, and especially Old Gamboa Road – where some individuals had relatively high proportions of assignment (*q* ranged from 0.76 to 0.92) to the subcluster that was least represented in any of the other populations (Fig. 2).

### Distance-based cluster analysis

**Figure 4:**
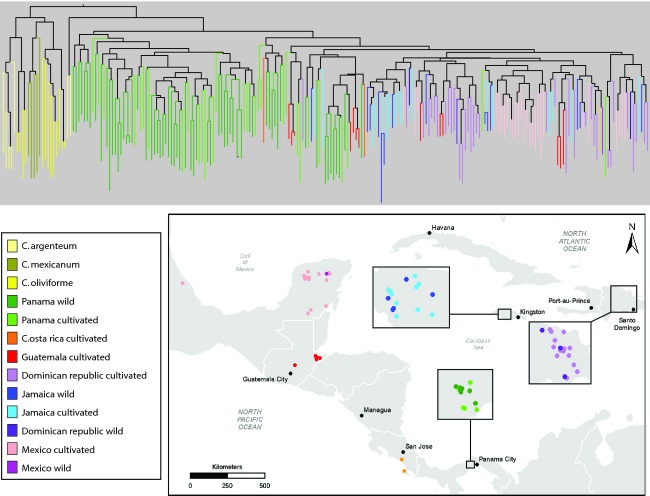
Neighbor-joining dendrogram constructed from Cavalli-Sforza's chord distances for all pairwise comparisons of individuals of *C. cainito* and close relatives based on genotypes at ten microsatellite loci. Branches are color-coded by species, and, within *C. cainito*, by geographic origin and cultivation status (see inset map), with darker shades of each color representing wild individuals and lighter shades representing cultivated individuals.

In the neighbor-joining (NJ) dendrogram, the sampled individuals generally formed species-unique clusters (Fig. 4). *Chrysophyllum oliviforme* formed two clusters. *Chrysophyllum mexicanum* formed one cluster that was nested within *C. oliviforme*. *Chrysophyllum argenteum* grouped principally into one cluster except for one locality from the Osa Peninsula (OSA, Costa Rica, Table 1), which was sister to all of the *C. cainito* individuals (Fig. 4).

Two principal groups were resolved within *C. cainito*, one including 65 of the 95 (68%) wild and cultivated trees collected in Panama, and a second that included the remaining wild and cultivated trees sampled from Panama as well as all of the trees sampled from Costa Rica, northern Mesoamerica (Guatemala, Mexico), and the Antilles (Dominican Republic and Jamaica). Within the second group, most (27 of 30, 90%) of the remaining trees from Panama fell into three clusters that formed successive sister groups to a large cluster including the vast majority of trees from the north, suggesting that the latter were derived from the former. Throughout the dendrogram, cultivated trees were largely intermixed with wild trees and neither was resolved as a distinct group, either across all sampling localities or within specific geographic regions.

### Partitioning of genetic structure

Three separate AMOVAs were conducted: one treating individuals from northern and southern localities, respectively, as separate populations, the second treating cultivated and wild trees from the north as separate populations, and the third treating cultivated and wild trees from the south as separate populations (Table 3). In all three cases, the greatest proportion of allelic variation (67% across all samples, and within the north, 90% within the south) was partitioned within individuals, reflecting high levels of heterozygosity and allelic richness across all loci, with higher levels of both in the south. The higher level of variation partitioned among individuals in the north (29%) than in the south (7%) presumably reflects greater geographic isolation of areas sampled in the north (Figs 3 and 4). Sixteen percent of the variation was partitioned between northern and southern localities, while only 4% was partitioned between wild and cultivated trees in the north, and only 2% was partitioned between wild and cultivated trees in the south. These results mirror those from the STRUCTURE and distance analyses in revealing relatively strong differentiation between trees from northern and southern localities and very little differentiation between wild and cultivated trees in either area.

**Table 3:** AMOVA partition for molecular variance in *Chrysophyllum cainito*. Molecular variance of *C. cainito* was partitioned among all collection localities, among northern and southern geographic regions, and separately for all wild and all cultivated tree localities. *P*-values were obtained after 9999 permutations. The degree of freedom (df), variance component, and percent variation for each AMOVA partition are provided.

Source of variation	df	Variance components	% variation	*P*-value
Northern and southern				
Among northern and southern regions	1	0.621	16	0.001
Among individuals within northern and southern regions	204	0.634	17	0.001
Within all individuals	206	2.587	67	0.001
Northern localities				
Among northern wild and northern cultivated localities	1	0.131	4	0.001
Among individuals within a northern locality	107	0.909	29	0.001
Within northern individuals	109	2.106	67	0.001
Southern localities				
Among southern wild and southern cultivated localities	1	0.074	2	0.001
Among individuals within a southern locality	95	0.256	7	0.002
Within southern individuals	97	3.129	91	0.001

## Discussion

### Provenance of wild and domesticated Chrysophyllum cainito

Both Bayesian (Figs 2 and 3) and distance-based (Fig. 4) cluster analyses separated the sampled individuals of *C. cainito* into two major groups, one comprised primarily of wild and cultivated individuals sampled from Panama in southern Mesoamerica, the other comprised primarily of individuals from the Antilles and northern Mesoamerica. Results from AMOVA mirrored those from the cluster analyses in revealing higher differentiation between trees from northern and southern localities than among individuals or between wild and cultivated trees within either area.

Our analyses further suggested that populations of *C. cainito* in northern Mesoamerica and the Antilles were derived from populations in southern Mesoamerica. Allelic richness and number of private alleles were significantly higher in the south than in the north, and the genetic diversity in the latter area represented a subset of that in the former. In the STRUCTURE analysis, more individuals collected from the south exhibited admixture with the primarily northern gene pool than the reverse, and two trees from Panama actually assigned highly to the primarily northern gene pool. Similarly, in the NJ tree based on genetic distances, the vast majority of trees from populations in the north formed a cluster nested within several clusters comprised of trees from populations in the south.

High levels of genetic admixture were observed across collecting localities within Panama (Fig. 2). These results are consistent with the general observation that tropical trees are highly outcrossing with high levels of gene flow (Ward et al. 2005). In contrast, four geographically structured gene pools, corresponding largely to country of collection, were inferred in the north. We hypothesize that these results, like those from AMOVA that revealed higher levels of variation partitioned among individuals in the north (29%) than in the south (7%), reflect greater geographic isolation of the areas sampled in the north, which may have resulted from founder effects that occurred as the species was dispersed across this region.

We hypothesize that *C. cainito* is native to the Isthmus of Panama and that the cultivated and wild trees in the Antilles and northern Mesoamerica were originally derived from populations on the Isthmus. We predicted that if caimito were native to the Antilles, cultivated trees would be derived from those wild populations and thus would harbor a moderate to high degree of the diversity present in the local wild populations. Our genetic data do not exhibit this pattern. Furthermore, we did not observe any trees in the Antillean and northern Mesoamerican populations that we sampled with putative undomesticated phenotypes like those observed in Panama (Parker et al. 2010). In Panama, the wild individuals we sampled were canopy-emergent trees, which were found in both primary forest and secondary forest and had fruit and seed centeracters that differed from cultivated trees, while in both the Dominican Republic and Jamaica, wild *C. cainito* trees were found only in disturbed areas, on edges of secondary forest, along roads, and along riparian areas, and they exhibited fruit and seed traits, as well as plant habit, similar to the domesticated trees in Panama (J. Petersen & D. Potter, pers. obs.). These results suggest that the wild trees in the northern part of the distribution are most likely secondarily wild, that is, escapes from cultivation or feral trees. We cannot rule out the possibility that ancestral, wild-type trees do or once did occur in these areas and are now rare because of frequent hurricanes or habitat conversion; we did not find them, and they have not been previously collected or reported, to our knowledge. Our observations are more consistent with the hypothesis that the forms that migrated into the Antilles were already semidomesticated. A similar pattern was reported in *Crescentia cujete* (jícara) where a unique domesticated form migrated via cultivation into the Yucatan Peninsula of Mexico (Aguirre-Dugua et al. 2012). These observations are also consistent with Miller and Knouft's study (2006), which indicated that the geographic range of a neotropical domesticate, *Spondias purpurea*, expanded under cultivation.

A Panamanian rather than Antillean origin of *C. cainito* is further supported by relationships with other species. A sample of *Chrysophyllum argenteum* subsp. *panamense*, native to Central and South America, was resolved as the closest relative of *C. cainito* in the NJ dendrogram (Fig. 4), and the same sample was shown in the STRUCTURE analysis to be genetically admixed with the southern *C. cainito* gene pool (Fig. 2). Similar relationships were revealed by DNA haplotype data (Petersen et al. 2012).

Taken together, our results suggest that caimito was brought into cultivation on the Isthmus of Panama and dispersed northward by humans to other parts of Mesoamerica and the Caribbean, and eventually to other areas in the New and Old World tropics, with accompanying reductions in genetic and phenotypic diversity. The data suggest that, rather than a single domestication in the Antilles, independent domestications in the north and south, or diffuse origins over a broad geographic range, there was a single domestication incorporating a broad sample of genotypes from wild populations within a relatively restricted geographic area in the region that is now Panama. These data are in agreement with our previous phylogeographic analysis that indicated one cultivated genotype dramatically increased under cultivation in Panama and spread northward through Mesoamerica and into the Antilles, which likely was mediated by human dispersal (Petersen et al. 2012). A similar pattern is observed in our microsatellite genotype data as illustrated in Fig. 3, where the percentage of the Gene Pool Cluster 1, which occurs at a low percentage in caimito trees collected in Panama, greatly increases in frequency in northern Mesoamerica, and the Greater Antilles, where it becomes the predominant gene pool.

Our hypothesis of a Panamanian origin of *C. cainito* refutes the prevailing view that the species is native to the Greater Antilles (e.g., Pennington 1990) and provides the first example we know of that implicates the Isthmus of Panama as a center of origin for a now widely cultivated crop species. Our conclusion that domestication of caimito took place within a single geographically restricted area provides a contrast to studies that have suggested that multiple and/or diffuse origins over a broad geographic range is a more common pattern for perennial species (Miller and Gross 2011). Other species that have been reported to have a single geographic origin of domestication include both temperate and tropical perennials such as grape (Near East), oil palm (West Africa), fig (Middle East/lower Jordan Valley), kiwifruit (China), clementine (northern Africa), and possibly cacao (Mesoamerica) (Clement et al. 2010; Miller and Gross 2011).

One caveat to our conclusion is that we did not include any samples of *C. cainito* from South America, thereby precluding our ability to test the possibility that the species is native there as well as in Panama. Our sampling of collection localities was determined by reviewing the literature and herbaria specimen data and by speaking with local flora experts in the Antilles, Central and South America to determine whether or not wild and/or cultivated trees of *C. cainito* were present. We focused our primary collecting efforts in the two areas where the species has been considered native according to published reports: the Greater Antilles and the Isthmus of Panama.

### Effects of human selection on levels of genetic diversity in cultivated caimito

Overall, cultivated individuals of caimito were slightly but not significantly less diverse than wild individuals. These results indicate that cultivated *C. cainito* did not experience a major bottleneck event in the initial events of artificial selection, a centeracteristic pattern for domesticated perennial species (reviewed by Pickersgill 2007; Miller and Gross 2011). Cultivated individuals of other neotropical tree fruit species also retain high levels of genetic diversity in comparison with wild progenitor populations. Jocote (*Spondias purpurea*) contained close to 90% of the wild diversity (Miller and Schaal 2006), and domesticated individuals of avocado (*Persea americana*) retained roughly 80% of the diversity present in wild progenitor populations (Chen et al. 2008). Cultivated trees of inga (*Inga edulis*) retained 80% of the allelic diversity of wild trees (Hollingsworth et al. 2005), and similar patterns were observed using chloroplast data (Dawson et al. 2008). Our results also fit the general observation, based on data from codominant neutral markers, that perennial fruit crops and their progenitors retain higher levels of genetic variation than their annual counterparts, 94.8% versus 59.9% on average, according to Miller and Gross (2011).

Additionally, our results provide a classic example of the lack of correspondence that is often observed between neutral genetic markers and phenotypic centeracters under selection (e.g., Papa et al. 2005; Sahli et al. 2008; Arraouadi et al. 2009; Rhone et al. 2010; Kawakami et al. 2011; Dutkowski and Potts 2012; see reviews in McKay and Latta 2002; Leionen et al. 2008). The microsatellite markers employed here evidently are not tracking the fruit and seed traits that were subject to strong human selection during domestication of caimito in Panama.

In the NJ dendrogram, caimito trees collected in Panama fall into several clusters, all but one of which include both cultivated and wild trees (Fig. 4). Thus, it appears that cultivated trees within Panama are derived from multiple wild populations, a conclusion also supported by the generally high levels of admixture across all wild and cultivated individuals in Panama observed in the STRUCTURE analysis (Fig. 2). We did, however, detect one distinct gene pool cluster among wild *C. cainito* trees in Panama, comprising individuals from the Pipeline Road, Venta de Cruces, and Old Gamboa sampling localities, that showed little admixture with the other three gene pools inferred for that region, indicating that some of the genetic variation in the wild is not well represented in cultivated material.

Cultivated individuals of caimito retain high levels of the diversity present in wild trees and reinforce the view that cultivated trees act as important reservoirs for genetic diversity, a fact that is particularly noteworthy because natural, wild populations are increasingly at risk due to fragmentation (e.g., Aldrich and Hamrick 1998; Fuchs et al. 2003) and overextraction (e.g., Newton 2008). Cultivated species fulfill multiple roles in small-scale landholdings in tropical areas. They are important sources of nutrition (Simpson and Ogorzaly 2001; Schreckenberg et al. 2006; Haq et al. 2008) and income in local economies, as well as providing a type of genetic refugium, thereby slowing the loss of biodiversity (see recent review by Dawson et al. 2013).

These results underscore the value of including phylogeographic analyses of genetic data well as morphological and ecological information in conservation efforts. The limitations of ex situ conservation strategies are exacerbated in cases such as caimito, which has a small, geographically restricted center of origin. Very few germplasm collections exist currently for this species, and material from Panama is poorly, if at all, represented in them (Gazel Filho 1995; Campbell et al. 2006, 2010; USDA-ARS 2012). Data on the center of origin of a species, as well as the areas where it exhibits greatest diversity, are essential for the successful design and implementation of both in situ and ex situ conservation programs.

The biota of neotropical forests are among the richest in the world. Future studies that examine the origins and diversity of semidomesticates in these regions will be important not only for expanding our understanding of evolution under domestication, but also for shedding light on long-and short-term processes that affect dynamic interactions between people and plants in these important ecosystems.
